# A Study on Trends in the Number and Content of Journal Article Titles Containing the Term “Elder Abuse”

**DOI:** 10.1093/geroni/igab046.2818

**Published:** 2021-12-17

**Authors:** Asako Katsumata, Noriko Tsukada

**Affiliations:** 1 Rehabilitation Institution, Chiba, Chiba, Japan; 2 Nihon University, Tokyo, Tokyo, Japan

## Abstract

Japan enacted the Elder Abuse Prevention Law on April 1, 2006; no amendments have been made since then. The purpose of this study is to examine trends in the number and content of journal article titles containing the term “elder abuse” and determine what further research is needed to identify where amendments to the law would be useful. We identified 986 articles using the CiNii database between the dates of 2005 to 2020. We categorized those titles by content, using a KJ Method. Preliminary analyses revealed that the average number of the articles published each year was 61.6, though a moving average of the numbers of articles on elder abuse has been steadily declining, as opposed to the average number for child abuse articles, 158, where the moving average staying the same. As for the analyses for the titles, they were categorized into 10 categories, including “law,” “responses of professional personnel to elder abuse,” “systems of government agencies,” “responses of nurses,” “institutional elder abuse,” “support for caregivers,” “reports on elder abuse in other countries,” “dementia and elder abuse,” “responses of medical institutions,” and “others.” It is suggested that more research needs to be done, especially in such areas as “verification of elder abuse cases,” “psychological impacts on elder abuse victims,” “empirical research conducted by medical doctors dealing with elder abuse cases,” and “cooperation between police and professional institutions,” many of which were found in research on child abuse where 4 law amendments have been made since its enactment of 2000.

